# Chromosome segregation occurs by microtubule pushing in oocytes

**DOI:** 10.1038/s41467-017-01539-8

**Published:** 2017-11-14

**Authors:** Kimberley Laband, Rémi Le Borgne, Frances Edwards, Marine Stefanutti, Julie C. Canman, Jean-Marc Verbavatz, Julien Dumont

**Affiliations:** 1 Institut Jacques Monod, CNRS, UMR 7592, University Paris Diderot, Sorbonne Paris Cité, 75205 Paris, France; 2 Department of Pathology and Cell Biology, Columbia University Medical Center, New York, NY 10032 USA; 3 Max Planck Institute of Molecular Cell Biology and Genetics (MPI-CBG), Dresden, 01307 Germany

## Abstract

During cell division, spindle microtubules ensure an equal repartition of chromosomes between the two daughter cells. While the kinetochore-dependent mechanisms that drive mitotic chromosome segregation are well understood, in oocytes of most species atypical spindles assembled in absence of centrosomes entail poorly understood mechanisms of chromosome segregation. In particular, the structure(s) responsible for force generation during meiotic chromosome separation in oocytes is unclear. Using quantitative light microscopy, electron tomography, laser-mediated ablation, and genetic perturbations in the *Caenorhabditis elegans* oocyte, we studied the mechanism of chromosome segregation in meiosis. We find spindle poles are largely dispensable, and in fact act as brakes for chromosome segregation. Instead, our results suggest that CLS-2-dependent microtubules of the meiotic central spindle, located between the segregating chromosomes and aligned along the axis of segregation, are essential. Our results support a model in which inter-chromosomal microtubules of the central spindle push chromosomes apart during meiotic anaphase in oocytes.

## Abstract (web summary)

In oocytes of most species atypical spindles assembled in the absence of centrosomes drive chromosome segregation, however the forces driving this process are unclear. Here the authors found that spindle poles are largely dispensable and that inter-chromosomal microtubules of the central spindle control chromosomal segregation.

## Introduction

Kinetochore-mediated attachment to spindle microtubules is a universal feature of mitotic chromosome segregation that is conserved in *Caenorhabditis elegans*. Dynamic microtubules that emanate from the centrosomes at the spindle poles are linked to kinetochores and generate pulling forces to drive poleward chromosome movement in anaphase A and to drag chromosomes during anaphase B spindle elongation^1^. In contrast to the canonical kinetochore-mediated pulling, a kinetochore-independent mechanism is responsible for chromosome segregation during female meiosis in *C. elegans* ^2,3^. Furthermore in *C. elegans* oocytes, microtubules at the spindle pole depolymerize substantially during metaphase and early anaphase, so that by anaphase onset, miniature spindle poles are almost directly adjacent to the poleward chromosomal surface^4^. An initial study proposed that, in *C. elegans* oocytes, functional spindle poles are dispensable for chromosome segregation and hypothesized instead the existence of an atypical central spindle-driven pushing mechanism^3^. However, recent contradictory studies implicated the microtubule minus-end directed motor dynein in a chromosome sliding model or a network of proteins comprised of the chromosome-bound severing protein Katanin (MEI-1/2 in *C. elegans*), the spindle pole protein ASPM-1^Asp^ and the microtubule-associated protein (MAP) LIN-5^NuMA^ in an atypical kinetochore-independent microtubule-based pulling model of meiotic chromosome segregation^5,6^. Importantly, and in contrast to the initial report, both models required the presence of functional spindle poles to relay the forces exerted by motors or MAPs to chromosomes, and to move them apart in anaphase. The nature of the machinery responsible for force generation during meiotic anaphase and the requirement for spindle poles during chromosome segregation in the *C. elegans* oocyte thus remain unclear.

Here we set out to determine the mechanism of meiotic chromosome segregation in oocytes. For this, we analyze with high-resolution the spatial and temporal organization of microtubules at high-resolution during meiotic anaphase in the *C. elegans* oocyte. We used electron tomography to analyze microtubule organization during meiotic anaphase in the *C. elegans* oocyte. We find that spindle poles and central spindle microtubules form two distinct anaphase arrays that do not significantly overlap. Furthermore, the central spindle is itself comprised of two overlapping arrays formed by short microtubules that either contact one set of chromosomes (the chromosomal array) or do not contact chromosomes (the central array). By performing laser-mediated ablation in live oocytes, we show that spindles poles are dispensable and instead act as brakes for chromosome segregation. In contrast, the central spindle is essential and provides the primary force for segregating the two sets of meiotic chromosomes by pushing them apart during anaphase. Functional analysis of the CLS-2^CLASP^ 3A mutant impaired in microtubule polymerase activity suggests that central spindle microtubules, essential for meiotic chromosome segregation, are assembled through the activity of CLS-2^CLASP^ ^7^. Thus unlike in most mitotic cells, chromosome segregation in *C. elegans* oocytes is powered by central spindle-mediated pushing forces between the separating chromosomes.

## Results

### Electron tomography of anaphase spindles in *C. elegans* oocytes

**Fig. 1:**
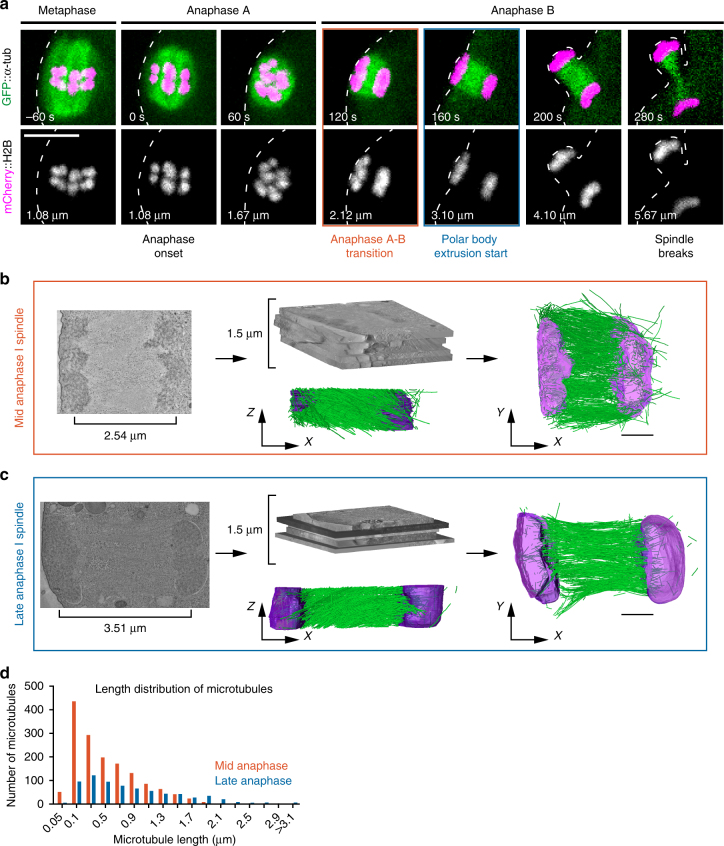
Three-dimentional reconstructions of the anaphase meiotic spindle by electron tomography. **a** Still images from live imaging of a fertilized oocyte expressing GFP-tagged tubulin and mCherry-tagged histone H2B. Timing from anaphase onset and the distance between the centroid of each set of segregating chromosomes are indicated at the bottom left corner of each frame. The two meiotic stages used for spindle reconstruction by tomography are framed in red (mid-anaphase) or blue (late anaphase). Scale bar, 5 µm. **b**, **c** Strategy used and partial spindle reconstructions for the mid- **b** and late **c** anaphase I spindle. (Left) *XY* plane image of the combined dual-axis tomogram. (Middle) Stitching of five successive 300 nm-thick serial sections used to generate the combined tomogram and *XZ* projections of the final reconstruction. Microtubules (green), chromosomes (magenta). (Right) *XY*-projected model of chromosomes (magenta) and microtubules (green). Scale bars, 1 µm. **d** Length distribution of fully contained microtubules for mid- (red) and late (blue) spindle reconstructions

To better understand the physical relationship between spindle microtubules and chromosomes during segregation in oocytes, we quantitatively analyzed the 3-dimentional organization of microtubules within the anaphase meiotic spindle by correlative light and electron tomography^8^. *Ex utero* fertilized oocytes expressing green fluorescent protein (GFP)-tagged tubulin and mCherry-tagged H2B were imaged using a spinning-disk microscope until they reached mid- or late anaphase I of meiosis (Fig. 1a). They were then fixed and processed for electron tomography. 300 nm-thick sections were cut mostly parallel to the chromosome segregation axis to ensure inclusion of both spindle poles, and a 1.5 μm-thick slab of each stage spindle was reconstructed (Fig. 1b, c).

In the earlier mid-anaphase I spindle, the centroid of the two sets of chromosomes were 2.54 μm apart which, as indicated by analysis of chromosome distance over time in live cell imaging experiments, corresponded to the anaphase A to B transition (or anaphase A-like to B-like transition, as both processes are kinetochore independent^3^, hereafter anaphase A and B; Fig. 1a, b, Supplementary Fig. 1a and Supplementary Movie 1). The mid-anaphase I reconstructed spindle consisted of 2880 microtubules of which 1514 (52.5%) were fully contained within the reconstructed slab (i.e., both plus and minus ends contained within the boundaries of the partial reconstruction, with the plus ends identifiable by the “open” or flared appearance at the tip^9^; Supplementary Fig. 1b). The second, late anaphase I spindle was imaged at the beginning of polar body extrusion about mid-way through anaphase B (when the chromosome centroids had separated by a distance of 3.51 μm; Fig. 1a, c, Supplementary Fig. 1a and Supplementary Movie 2). This late anaphase I reconstruction contained 1310 microtubules of which 722 (55%) were fully contained within the reconstruction (Supplementary Fig. 1b). The length distribution of fully contained microtubules peaked at relatively short lengths (<500 nm) for both mid- and late anaphase I spindles (Fig. 1d). The late anaphase I spindle contained fewer but longer microtubules, suggesting that individual meiotic spindle microtubules either disassemble or elongate over time during anaphase I. Importantly the 26% reduction in the total combined length of microtubules observed in the two reconstructions (1499 vs. 1102 μm in mid- and late anaphase respectively) was in full agreement with the 25% fluorescence intensity drop observed on average between these two stages by live imaging of fluorescently-tagged microtubules (0.28 vs. 0.21 a.u. in mid- and late anaphase respectively, Supplementary Fig. 1b–d). Our two spindle reconstructions therefore demonstrate a representative view of microtubule populations at these stages of anaphase.

**Fig. 2:**
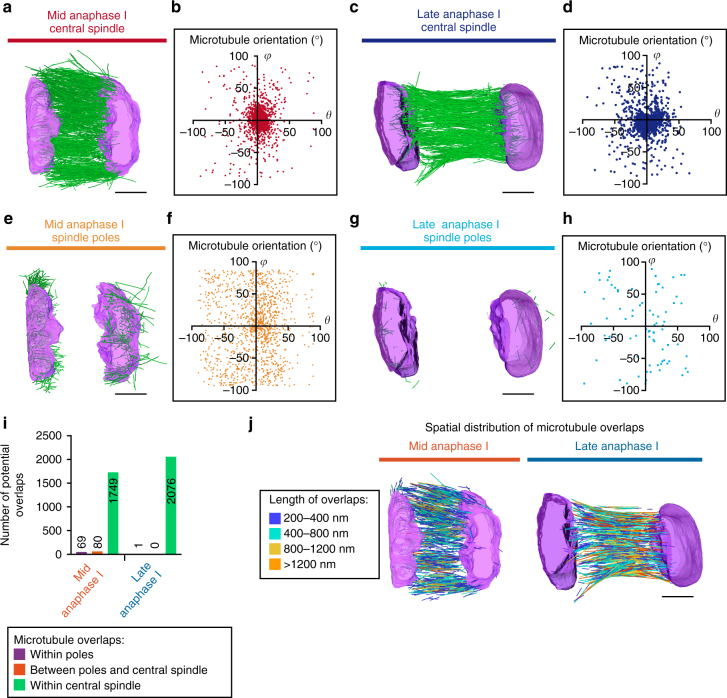
Meiotic spindle poles are comprised of randomly organized short microtubules. **a**, **c** Central spindle microtubule population in the mid- **a** or late **c** anaphase I reconstructions. **b**, **d** Distribution of microtubule inclination (*θ*) and azimuthal (*φ*) angles for the mid- **b** or late **d** anaphase I central spindle microtubules. **e**, **g** Spindle pole microtubule population in the mid- **e** or late **g** anaphase I reconstructions. **f**, **h** Distribution of microtubule inclination (*θ*) and azimuthal (*φ*) angles for the mid- **f** or late **h** anaphase I spindle pole microtubules. **i** Quantification of microtubule overlaps >200 nm in length for the indicated populations. **j** Visualization of the spatial distribution of microtubule overlaps >200 nm in length in mid- and late anaphase I spindles. The length of overlaps is color-coded as indicated. Scale bars, 1 µm

Two populations of microtubules were evident in both tomograms: one inside the chromosomes, and one on the outward-facing poleward side of the chromosomes. The first population, which represented the majority of microtubules (1655 and 1229 microtubules in mid- and late anaphase I respectively), was aligned along the chromosome segregation axis in an anaphase central spindle-like organization (termed hereafter central spindle), contained between the two sets of segregating chromosomes (Fig. 2a, c and Supplementary Fig. 1b)^10^. The second population with random orientation was comprised of 1225 and (only) 81 microtubules in mid- and late anaphase I respectively, and concentrated on the poleward-facing surface of chromosomes (termed hereafter spindle poles), and thus likely correspond to disassembling spindle pole remnants (Fig. 2e, g)^4^. To quantitatively analyze microtubule orientation within each population in the mid- and late anaphase reconstructions, we measured the inclination (*θ*) and azimuthal angles (*φ*) of individual microtubules relative to the chromosome segregation axis. More than 80% of central spindle microtubules between the separated chromosomes clustered together with *θ* and *φ* values comprised between −30° and +30° from both reconstructed spindles (86.1 and 81.1% microtubules in mid- and late anaphase I spindles respectively; Fig. 2b, d). These microtubules are thus directed along the chromosome segregation axis and organized either parallel or antiparallel to each other. In contrast, microtubules that formed the pole remnants on the outward-facing sides of the chromosomes were spread over the polar plots at both stages (31.8 and 25.3% microtubules in mid- and late anaphase I spindles respectively with *θ* and *φ* between −30° and +30°), consistent with a more random orientation of these microtubules (Fig. 2f, h). These observations demonstrate that spindle pole remnants, present at these two stages, are not specifically oriented perpendicular to the chromosome surface as would be expected for classical functional spindle poles that drive chromosome segregation. The reduced proportion of spindle pole microtubules in the late anaphase reconstruction also shows that spindle pole remnants are disassembling between the anaphase A to B transition and the beginning of polar body extrusion. To test for direct interactions between the spindle pole and central spindle microtubules, as predicted by a pole-based chromosome segregation model, we analyzed the number and distribution of potential functional microtubule overlaps^6^. For this, we considered pairs of microtubules with a lateral distance of <45 nm, which is similar to the observed distance between overlapping microtubules in tomographic reconstructions of yeast and mammalian mitotic spindles^11,12^. To exclude microtubules that intersect each other at steep angles, which will appear as overlapping over short distances at the site of crossing, we only considered overlaps ≥200 nm in length (Methods section). The vast majority of potential overlaps observed were between individual central spindle microtubules in both mid- and late anaphase I reconstructions (92.1 and 99.9% in mid- and late anaphase I spindles respectively; Fig. 2i, j). Together, these results do not support a mechanical connection between spindle pole remnants and central spindle microtubules during anaphase I in the *C. elegans* oocyte.

### The central spindle contains two distinct microtubule arrays

**Fig. 3:**
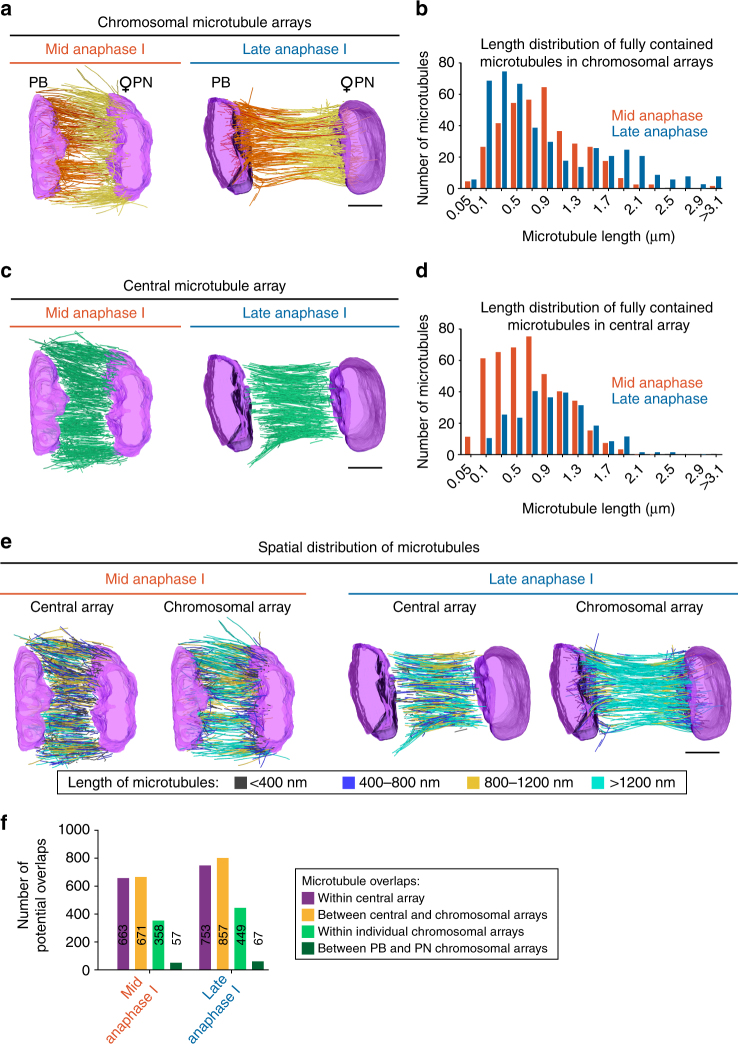
The meiotic central spindle is comprised of two interconnected arrays of microtubules. **a** Partial spindle reconstructions for mid- (left) and late (right) anaphase I chromosomal microtubule arrays. Model of chromosomes (magenta) and either polar body (PB) proximal microtubules (orange), future maternal pronucleus (PN) proximal microtubules (yellow), or microtubules which contact both chromosome masses (red). **b** Length distribution of fully contained microtubules in the chromosomal microtubule arrays for mid- (orange) and late (blue) anaphase I spindle reconstructions. **c** Partial spindle reconstructions for mid- (left) and late (right) anaphase I central microtubule arrays. Model of chromosomes (magenta) and microtubules (green). **d** Length distribution of fully contained microtubules in the central array for mid- (orange) and late (blue) anaphase I spindle reconstructions. **e** Microtubule length distribution within partial spindle reconstructions for mid-anaphase I central (far left) and chromosomal (left) microtubule arrays, and late anaphase I central (right) and chromosomal (far right) microtubule arrays. Microtubules are color-coded according to their length as indicated. Scale bars, 1 µm. **f** Quantification of microtubule overlaps >200 nm in length for the indicated populations

Analysis of microtubule ends within the central spindle identified two major sub-populations or arrays and revealed that very few microtubules were in contact with both sets of segregating chromosomes (12 and 6 in mid- and late anaphase I spindles respectively; Supplementary Fig. 1b). The first sub-population had at least one microtubule end in direct contact with one set of the segregating chromosomes (termed the chromosomal arrays hereafter; Fig. 3a). The second sub-population formed a central array of microtubules aligned along the axis of segregation with neither end of the microtubules in contact with the chromosomes (termed the central array hereafter; Fig. 3c). Analysis of the spatial distribution of microtubule lengths revealed that the central array formed a tiled network of microtubules with no preferential length-dependent targeting in either mid- or late anaphase I central spindles (Fig. 3e). Microtubule length distribution was consistent with longer microtubules present in the late anaphase I central spindle in both types of microtubule arrays (Fig. 3b, d and Supplementary Fig. 1e, f). Chromosomal and central arrays of microtubules were also both closely interconnected as demonstrated by the large proportion of potential microtubule overlaps observed between the two populations of microtubules at both stages (Fig. 3f). Potential overlaps were distributed all over the volume of the central spindle with no observable preferential local concentration (Fig. 2j). Thus, the tiled central array is comprised of microtubules with an average length of 665 and 956 nm in mid- and late anaphase I spindles respectively, and links the two chromosomal arrays (with an average microtubule length of 508 and 848 nm in mid- and late anaphase I spindles respectively) through multiple overlap zones. These results demonstrate that the oocyte meiotic central spindle is comprised of two interconnected sub-populations (or arrays) of microtubules that grow during the process of chromosome segregation.

### Spindle poles are dispensable for chromosome segregation

**Fig. 4:**
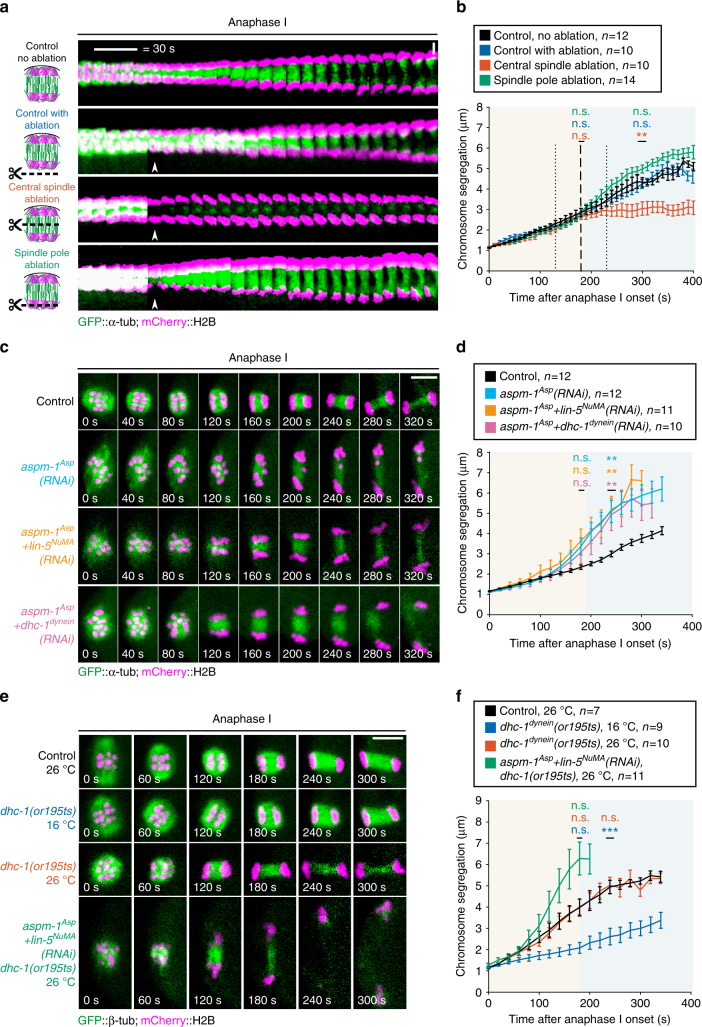
The spindle poles act as brakes during meiotic chromosome segregation. **a** Schematics and still images from live imaging of control not ablated (top), control ablated (second from top), central spindle (second from bottom) and spindle pole (bottom) laser-mediated ablation in GFP-tagged α-tubulin and mCherry-tagged histone H2B expressing fertilized oocytes. White arrowheads indicate the ablation events. Scale bar, 2 µm. **b** Distance between the centroid of each set of segregating chromosomes over time after anaphase onset in the indicated conditions. Error bars represent the SEM. Thick and thin vertical dashed lines represent the average and minimum/maximum times of ablation respectively. Background colors represent average duration of anaphase A (light orange) and B (light blue) respectively. (One-way ANOVA: control ablation at 180 s, *p* = 0.9297; central spindle at 180 s, *p* = 0.9007; Spindle pole at 180 s, *p* = 0.9924; control ablation at 300 s, *p* = 0.9951; central spindle at 300 s, *p* = 0.0045; spindle pole at 300 s, *p* = 0.1771) **c** Still images from live imaging of fertilized oocytes expressing GFP-tagged α-tubulin and mCherry-tagged histone H2B in the indicated conditions. Timings from anaphase onset are indicated at the bottom of each frame. Scale bar, 5 µm. **d** Distance between the centroid of each set of segregating chromosomes over time after anaphase onset in the indicated conditions. Tracking was performed until the anaphase spindle broke apart at the end of polar body extrusion. Error bars represent the SEM. Background colors represent average duration of anaphase A (light orange) and B (light blue) respectively. (One-way ANOVA: *aspm-1* ^*Asp*^ *(RNAi)* at 180 s, *p* = 0.2281; *aspm-1* ^*Asp*^ *+ lin-5* ^*NuMA*^ *(RNAi)* at 180 s, *p* = 0.0910; *aspm-1* ^*Asp*^ *+ dhc-1* ^*dynein*^ *(RNAi)* at 180 s, *p* = 0.6103; *aspm-1* ^*Asp*^ *(RNAi)* at 240 s, *p* = 0.0018; *aspm-1* ^*Asp*^ *+ lin-5* ^*NuMA*^ *(RNAi)* at 240 s, *p* = 0.0015; *aspm-1* ^*Asp*^ *+ dhc-1* ^*dynein*^ *(RNAi)* at 240 s, *p* = 0.0077) **e** Still images from live imaging of fertilized oocytes expressing GFP-tagged β-tubulin and mCherry-tagged histone H2B in the indicated conditions. Timings from anaphase onset are indicated at the bottom of each frame. Scale bar, 5 µm. **f** Distance between the centroid of each set of segregating chromosomes over time after anaphase onset in the indicated conditions. Tracking was performed until the anaphase spindle broke apart at the end of polar body extrusion. Error bars represent the SEM. Background colors represent average duration of anaphase A (light orange) and B (light blue) respectively. (One-way ANOVA: *dhc-1(or195ts)*, 16 °C at 180 s, *p* = 0.0926; *dhc-1(or195ts)*, 26 °C at 180 s, *p* = 0.9999; *aspm-1* ^*Asp*^ *+ lin-5* ^*NuMA*^ *(RNAi) + dhc-1(or195ts)*, 26 °C at 180 s, *p* = 0.4751; *dhc-1(or195ts)*, 16 °C at 240 s, *p* = 0.0009; *dhc-1(or195ts)*, 26 °C at 240 s, *p* = 0.9853; *aspm-1* ^*Asp*^ *+ lin-5* ^*NuMA*^ *(RNAi) + dhc-1(or195ts)*, 26 °C at 240 s not available)

To determine the function of spindle poles and the central spindle during meiotic chromosome segregation, we performed a set of laser ablation experiments in dividing oocytes expressing GFP-tubulin and mCherry-histone H2B. We first demonstrated the efficiency of our ultraviolet (UV)-pulsed laser ablation approach by targeting the central spindle microtubule population at the anaphase A to B transition (Fig. 4a). Indeed, in all three models of meiotic chromosome segregation, the central spindle plays a key role and is required for chromosome separation. Accordingly, ablating central spindle microtubules at the anaphase A to B transition led to a complete block in the segregation process within seconds of the ablation event (Fig. 4b). The inhibition of chromosome segregation observed in this condition was not caused by an off-target effect of the laser ablation itself as targeting an area outside of the spindle did not have any effect on the rate of chromosome segregation. We then tested if spindle pole remnants present at this stage are also required for meiotic chromosome segregation. For this we performed a targeted ablation of the spindle pole associated with the oocyte interior chromosomes that are maintained by the oocyte to form the future maternal pronucleus. The outer spindle pole and associated chromosomes are in close proximity to the plasma membrane, which precluded ablating that spindle pole without wounding the oocyte. Strikingly, laser ablation of the spindle pole did not block chromosome segregation as was observed after central spindle ablation (Fig. 4a). Instead, chromosome segregation persisted as in control non-ablated oocytes (Fig. 4b). Overall, these results show that the central spindle is essential for meiotic chromosome segregation in the *C. elegans* oocyte but that the integrity of spindle poles is not.

The pole-based pulling force model for meiotic chromosome segregation proposes that non-canonical microtubule pulling forces from the chromosomes to the spindle poles drive chromosome movement^6^. Thus, to further probe the function of the spindle poles in oocytes, we analyzed meiotic chromosome segregation after spindle pole disruption by RNA interference (RNAi)-mediated depletion of the microtubule minus-end protein ASPM-1^Asp^ alone or in combination with LIN-5^NuMA^ ^13–15^. Despite the abnormally long spindles with unfocused and disorganized spindle poles, neither ASPM-1^Asp^ alone nor ASPM-1^Asp^ and LIN-5^NuMA^ co-depletion blocked chromosome segregation in anaphase I (Fig. 4c and Supplementary Fig. 2a and Supplementary Movie 3). Chromosome tracking over time in these pole-disrupted oocytes revealed that the rate of chromosome separation during anaphase A was initially comparable to controls (Fig. 4d). However, it became significantly faster at the anaphase A to B transition, which ultimately led to a greater extent of chromosome segregation over time. These results show that properly organized spindle poles are not required for chromosome segregation in *C. elegans* oocytes. In contrast, the faster separation observed in absence of organized spindle poles and in the ablation experiment suggests that spindle poles may instead act as brakes during meiotic chromosome segregation.

### Dynein is dispensable for chromosome segregation

Another model of meiotic chromosome segregation proposes that dynein-dependent sliding forces move chromosomes apart along central spindle microtubules^5^. The unfocused spindle poles observed in ASPM-1^Asp^ and LIN-5^NuMA^-depleted oocytes are reminiscent of dynein inhibition in most cell types^16,17^. This prompted us to analyze the dynamic localization of dynein in these oocytes. In control oocytes, dynein concentrates on the external surface of chromosomes during prometaphase I and to the spindle poles throughout meiosis I^13^. In contrast and consistent with the unfocused pole phenotype, in ASPM-1^Asp^ and LIN-5^NuMA^-depleted oocytes, dynein was absent from chromosomes and spindle poles throughout anaphase B (Supplementary Fig. 2b, c and Supplementary Movie 4). To further test if kinetochore-localized dynein is important for chromosome segregation, we analyzed meiotic chromosome segregation after RNAi-mediated depletion of ZWL-1^Zwilch^ (member of the RZZ kinetochore complex) or the kinetochore dynein adapter protein SPDL-1^Spindly^, and in a dynactin subunit *dnc-6* ^*p27*^ null mutant (Supplementary Fig. 3a–d and Supplementary Movie 5). All three proteins are essential for targeting dynein at the kinetochore but no obvious spindle defects were visible and meiotic chromosome segregation proceeded at a normal or faster rate than in controls^18–20^. Next, to directly assess the function of dynein during meiotic chromosome segregation without compromising metaphase spindle assembly, we used an available fast-acting temperature sensitive *(ts)* partial loss-of-function mutant of the dynein heavy chain DHC-1 (*dhc-1 (or195ts)*, hereafter *dynein(ts)*). At the restrictive temperature (26 °C), *dynein(ts)* mutant zygotes displayed the characteristic dynein partial loss-of-function mitotic spindle positioning defect and a lack of mitotic anaphase spindle oscillations (Supplementary Fig. 3e). However, meiotic chromosome segregation occurred successfully in both *dynein(ts)* and control oocytes at 26 °C. We then combined RNAi-mediated depletion of ASPM-1^Asp^ with a partial depletion of dynein in control oocytes or RNAi-mediated simultaneous depletions of ASPM-1^Asp^ and LIN-5^NuMA^ in *dynein(ts)* oocytes at 26 °C. In both conditions, meiotic chromosome segregation occurred and chromosome separation was similar to that in ASPM-1^Asp^-depleted oocytes (Fig. 4c–f and Supplementary Movie 6). Altogether, these results are not consistent with a dynein-dependent sliding model for meiotic anaphase B chromosome segregation^5^.

### CLS-2^CLASP^ activity is essential for chromosome segregation

**Fig. 5:**
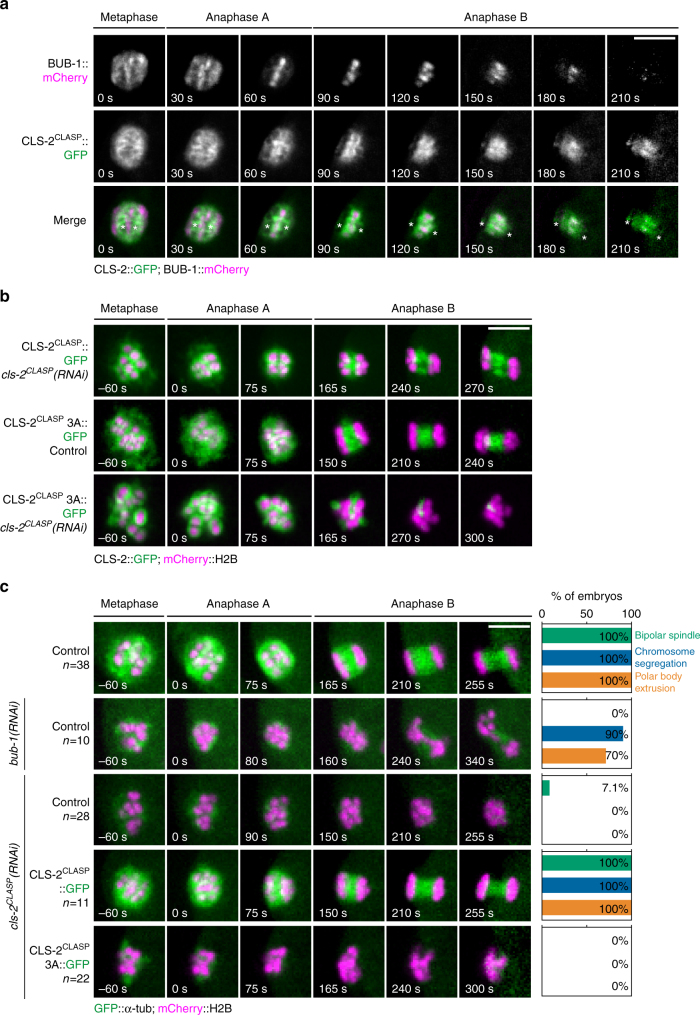
CLS-2^CLASP^ microtubule polymerase activity is required for meiotic chromosome segregation. **a** BUB-1::mCherry (top row), CLS-2^CLASP^::GFP (middle row), and merged (bottom row) localization during anaphase I. Asterisks denote the approximate centroid locations of the segregating chromosome masses. **b** GFP-tagged CLS-2^CLASP^ and CLS-2^CLASP^ 3A localization during anaphase I in the indicated conditions. **c** Still images from live imaging of fertilized oocytes expressing GFP-tagged α-tubulin and mCherry-tagged histone H2B in the indicated conditions. Quantifications of the percentage of observed bipolar spindles, chromosome segregation events, and polar body extrusion in meiosis I is shown on the right of each still sequence. Timings are indicated in the bottom left corner of each frame. Scale bars, 5 µm

Our data are instead consistent with meiotic anaphase B chromosome segregation being driven by central spindle microtubule-mediated pushing. We thus sought to identify the molecular mechanism that drives the assembly of central spindle microtubules at the anaphase A to B transition. During mitosis in *C. elegans* zygotes, central spindle microtubules assemble at anaphase onset through the activity of the MAP CLS-2^CLASP^ ^7^. Indeed, we previously showed that CLS-2^CLASP^ has microtubule polymerase activity that is essential for mitotic central spindle assembly. We thus hypothesized that this activity of CLS-2^CLASP^ could also be responsible for assembling meiotic central spindle microtubules^7^. Previous observations in fixed *C. elegans* oocytes demonstrated that CLS-2^CLASP^ along with its binding partners HCP-1/2^CENP-F^ and BUB-1 localize to the cup-like kinetochores and to ring-shaped structures surrounding the chromosomes, and then concentrate in stripes on the central spindle between the segregating chromosomes after anaphase onset^3^. Consistent with these previous observations in fixed oocytes, live imaging during meiosis I revealed that concomitant to the kinetochore disassembly which occurs after anaphase onset in *C. elegans* oocytes, CLS-2^CLASP^::GFP and BUB-1::mCherry progressively disappeared from the cup-like kinetochores and localized between the segregating chromosomes (Fig. 5a). During this phase, CLS-2^CLASP^::GFP surrounded BUB-1::mCherry and formed two stripes on the two inner faces of the segregating chromosomes. BUB-1::mCherry concentrated in microtubule poor regions, previously described as microtubule-free channels, that were particularly visible in end-on views of our mid-anaphase I spindle reconstruction (Supplementary Movie 1)^5^. CLS-2^CLASP^::GFP concentrated in close proximity to the ends of the two microtubule chromosomal arrays that we identified in both tomographic reconstructions. Thus, the localization of CLS-2^CLASP^ during anaphase of meiosis is consistent with a role for this microtubule polymerase in generating the microtubules responsible for the central spindle-mediated chromosome pushing forces in meiosis.

To determine the contribution of CLS-2^CLASP^ microtubule polymerase activity in meiotic chromosome segregation, we analyzed the localization and function of an RNAi-resistant CLS-2^CLASP^ mutant (CLS-2^CLASP^ W57A-K177A-R224A, hereafter CLS-2^CLASP^ 3A), which has impaired microtubule polymerase activity and prevents mitotic central spindle assembly^7^. CLS-2^CLASP^ 3A localized properly at the cup-like kinetochores, at the ring domains, and at the anaphase chromosomal stripes in presence or absence of endogenous CLS-2^CLASP^ during meiosis I (Fig. 5b). Expression of the CLS-2^CLASP^ 3A mutant following endogenous CLS-2^CLASP^ depletion severely disrupted meiotic spindle assembly and chromosome segregation and thus recapitulated the *cls-2* ^*CLASP*^ *(RNAi)* phenotype (Fig. 5c and Supplementary Movie 7). Importantly, the significant pre-anaphase spindle defects and overall reduction in microtubule density observed in both *cls-2* ^*CLASP*^ *(RNAi*) and CLS-2^CLASP^ 3A mutant oocytes was also observed following depletion of the CLS-2^CLASP^ upstream partner, BUB-1 (Supplementary Fig. 4a). However, BUB-1 depletion did not prevent meiotic chromosome segregation although lagging and stretched chromosomes were evident during anaphase (Fig. 5c)^3^. Thus, the lack of chromosome segregation in *cls-2* ^*CLASP*^ *(RNAi)* and CLS-2^CLASP^ 3A mutant oocytes cannot solely be explained by pre-anaphase spindle defects. Together, these results suggest that central spindle microtubule assembly in the *C. elegans* oocyte requires CLS-2^CLASP^ polymerase activity. Consistent with this model, we found that despite the loss of CLS-2^CLASP^ localization in BUB-1 depleted oocytes in metaphase, CLS-2^CLASP^::GFP still localized to central spindle microtubules in anaphase although to lesser extent than in controls (Supplementary Fig. 4b–d). This result suggests that CLS-2^CLASP^ localization on central spindle microtubules can occur independently of BUB-1 and does not strictly require prior metaphase localization of CLS-2^CLASP^ to meiotic kinetochores.

Together our results are consistent with a model of meiotic chromosome segregation in the *C. elegans* oocyte that involves outward pushing forces exerted by central spindle microtubules on chromosomes. Furthermore, the tiled array and high proportion of overlapping microtubules within the central spindle is compatible with an active process generating these overlaps and driving microtubule sliding. Interestingly, although the canonical mitotic central spindle motors and MAP also localize to the meiotic central spindle, including the kinesin-5 BMK-1^Eg5^, the kinesin-6 ZEN-4^MKLP1^, and the microtubule-bundling protein SPD-1^PRC1^, they do not positively contribute to meiotic chromosome separation during anaphase B (Supplementary Fig. 5a, b and Supplementary Movie 8)^6^. We thus propose that a combination of CLS-2^CLASP^-dependent microtubule assembly and central spindle motor and/or MAP-mediated microtubule sliding of the two anaphase chromosomal arrays (and potentially also the central arrays) are the primary forces driving chromosome segregation in this system.

## Discussion

Our direct measurement of the average microtubule and overlap lengths within each microtubule array is consistent with 6/7 overlapping microtubules linking the two sets of segregating chromosomes (Supplementary Fig. 5c). The exact nature of the microtubule motors and/or MAPs involved in generating the overlap and outward pushing forces at this stage remains unclear, but the plus-end directed motor kinesin-12 KLP-18^XKLP2^ and its interacting protein MESP-1 (MEiotic SPindle 1) are likely candidates^6,21,22^. Unfortunately, the strong metaphase monopolar spindle phenotype associated with depletion of either of these proteins in *C. elegans* oocyte precludes testing this hypothesis directly^23^. We favor KLP-18^XKLP2^ for several reasons. First, RNAi-mediated KLP-18^XKLP2^ depletion blocks meiotic chromosome segregation. Second, KLP-18^XKLP2^ concentrates between the segregating chromosomes, and more specifically in the region of the chromosomal microtubule arrays, during meiotic anaphase^23^. Third, the significant metaphase spindle defects associated with depletion of several ring domain components (BUB-1 and HCP-1/2^CENP-F^) does not prevent anaphase chromosome segregation^3^. Thus the complete lack of anaphase separation in absence of KLP-18^XKLP2^ cannot solely be explained by the pre-anaphase monopolar spindle phenotype, and may represent an active function of KLP-18^XKLP2^ at this stage. Testing this model will require tightly controlled temporal inactivation, which is not yet available for KLP-18^XKLP2^.

Central spindle-driven chromosome segregation may be generally relevant to cell division or may represent an adaptation specific to oocyte division in *C. elegans*. The extreme asymmetry of the divisions that lead to polar body extrusion in oocytes of most species is thought to be crucial for preserving cytoplasmic maternal stores essential for early embryo development^24^. The atypical spindle pole shrinkage that occurs during metaphase and early anaphase in *C. elegans* oocytes coupled to central spindle-driven segregation could help minimizing polar body volumes. Central spindle-driven segregation may also represent a general mechanism that contributes to chromosome segregation in other contexts. Parallel dominant mechanisms, including kinetochore-mediated pulling, may have concealed the existence of outward central spindle-mediated pushing forces in other cell types. Consistent with this view, during mitosis in the *C. elegans* zygote, chromosomes separate into two distinct masses with robust microtubule bundles present between them even in absence of functional centrosomes^25^. The poleward motion of kinetochore-less acentric chromosome fragments observed in insect spermatocytes could also suggest the existence of an outward kinetochore-independent central-spindle-driven pushing force in this system^26^. As central spindle microtubules are a common feature of anaphase spindles of most species, it will be interesting to test whether kinetochore-independent outward pushing forces play a conserved role in meiotic and mitotic chromosome segregation.

## Methods

### *C. elegans* strains and RNA-mediated interference

*C. elegans* strains used in this study are listed in Supplementary Table 1. All strains except JCC912 (maintained at 16 °C) were maintained at 23 °C on standard nematode growth media plates seeded with OP50 bacteria. The GCP485 strain (*dnc-6* ^*p27*^ *(−/−)*) was maintained using a GFP-marked balancer. F1 *dnc-6* ^*p27*^ *(−/−)* progeny from balanced heterozygous mothers were identified by the lack of GFP fluorescence^18^. The different lines were obtained either by stable single insertion by MosSCI or by crossing with pre-existing strains^27^. For RNAi experiments, double-stranded RNA (dsRNA) were produced by using the primers listed in Supplementary Table 2 to amplify regions from N2 genomic DNA, N2 cDNAs, or specific cDNAs. PCR reactions were cleaned (QIAGEN GmbH) and used as templates for 25 µL T3 and T7 transcription reactions (Ambion), which were combined and cleaned using an RNeasy kit (QIAGEN). Each RNA was eluted with 50 µL of elution buffer, then combined and mixed with 50 µL of nuclease-free H_2_O. Final dsRNA was annealed by incubating at 68 °C for 10 min followed by 37 °C for 30 min. For all dsRNAs except *dhc-1* ^*dynein*^, L4 hermaphrodites were injected with dsRNA and incubated at 20 °C for 44–48 h. *dhc-1* ^*dynein*^ *RNAi* was injected into adult worms 24 h after the *aspm-1* ^*Asp*^ *RNAi* injection.

### Live imaging and laser ablation

Fertilized oocytes were dissected and mounted in 5 μl of L-15 blastomere culture medium (0.5 mg/mL Inulin; 25 mM HEPES, pH 7.5; 3 mL Leibowitz L-15 Media and 1 mL Heat-Inactivated FBS per 5 mL total volume) on 24 × 60 mm coverslips sealed onto a metal holder. To prevent compression and evaporation, vaseline was used as a spacer and an 18 × 18 mm coverslip placed on top. Live imaging was done using a ×60/NA1.4 oil objective on a spinning-disk confocal microscope (Roper Scientific) and acquisition parameters were controlled by MetaMorph 7 software (Molecular Devices). For oocytes expressing α-Tubulin::GFP and mCherry::H2B (JDU233), 4 z sections every 2 µm were acquired at 20 s intervals. For oocytes expressing DHC-1^dynein^::GFP and mCherry::H2B (OD203), 4 z sections every 2 µm were acquired at 20 s intervals. For images presented in Fig. 5, JDU233, JDU205, and JDU282 were acquired with 4 z sections every 2 µm at 15 s using a ×60/NA1.4 oil objective with 2 × 2 binning. JDU107 was acquired with 2 z sections every 1 µm at 10 s interval using a ×60/NA1.4 oil objective. JDU146 was acquired with 2 z sections every 2 µm at 2 s interval using a ×60/NA1.4 oil objective. For experiments performed in JCC912 (*dynein(ts)*), worms were dissected at room temperature in cold medium. Imaging at 16 or 26 °C was performed using the CherryTemp temperature controller system (CherryBiotech, Rennes, France). For the laser ablation experiments, 4 z sections every 2 µm were acquired at 10 s intervals until the desired stage (anaphase A to B transition). Ablation was done using the iLas Pulse system (Roper Scientific), which corresponds to a Q-switched 355 nm UV-pulsed laser with a repetition rate of 6 kHz mounted on a spinning-disk microscope. 350 repetitions of UV pulse were applied to a 1-pixel wide line drawn perpendicular to the innermost pole, to the central spindle axis or outside of the spindle. For all live imaging experiments, maximal projections are presented. All kymographs were manually generated using Illustrator (Adobe) and Fiji softwares^28^.

### Live imaging analysis

Chromosome segregation was assessed by measuring the distance between segregating chromosome masses centroid over time using the Fiji software. Spindle pole focusing was evaluated by comparing the width of the metaphase I spindle poles to the central region of the spindle. Spindle pole and center widths were extracted from 3-pixel wide linescans drawn perpendicular to the 2 spindle poles and to the center of the spindle in Fiji. The average width of the 2 spindle poles was divided by the width of the spindle center to calculate a pole focusing score. Focused poles have a lower score than unfocused poles. Fluorescence intensity of GFP-tagged tubulin was measured over time as the integrated intensity in a 65 × 65 pixel rectangle that surrounded the spindle (IntDen(spin)). The same rectangle was drawn in the oocyte cytoplasm to measure average background at each time point (Mean(bck)). The corrected intensity of GFP-tagged tubulin was calculated as IntDen(spin)/Mean(bck). It was plotted over time after normalization to set minimum and maximum values to 0 and 1 respectively. Fluorescence intensity of GFP-tagged dynein was measured in a GFP-tagged DHC-1 (Dynein Heavy Chain) transgenic strain. A 20 × 20 pixel rectangle was drawn around a half spindle over time on the sum projections of a 4 z sections spaced by 2 µm stack and the integrated fluorescence intensity was extracted (IntDen(spin)). The same rectangle was drawn in the oocyte cytoplasm to measure background at each time point (IntDen(bck). The corrected intensity of GFP-tagged DHC-1^dynein^ was calculated and plotted over time as ((IntDen(spin)-IntDen(bck))/IntDen(bck). The same approach was used in Supplementary Fig. 4a to quantify GFP::tubulin and CLS-2::GFP intensities in metaphase and anaphase meiotic spindles, except a 55 pixel-diameter circle centered over the spindle was used for GFP::tubulin in metaphase, and 15 × 30 pixel rectangles were used for GFP::tubulin and CLS-2::GFP in anaphase.

### Correlative light and electron tomography and image processing

Wild-type *C. elegans* hermaphrodites expressing GFP-tagged β-tubulin and mCherry-tagged H2B were dissected in meiosis medium. Single meiosis I fertilized oocytes were transferred and enclosed in cellulose capillary tubes with 200 μm inner diameter (Leica Microsystems). Capillary tube-enclosed oocytes were then transferred in a drop of meiosis medium on a coverslip and observed with a spinning-disk confocal microscope using a ×60/NA0.7 air objective until mid- or late anaphase I of meiosis. They were then transferred into 1.5 × 0.1 mm high-pressure freezing membrane carriers (Leica Microsystems, Austria) filled with 1% BSA (Bovine Serum Albumin) in meiosis medium and cryo-immobilized within 30 s using an EM-PACT2 high-pressure freezer with a Rapid Transfer System (Leica Microsystems, Austria)^8,29^. Frozen samples were substituted in 1% OsO_4_, 0.1% Uranyl Acetate in Acetone using an automatic freeze substitution machine (EM AFS, Leica Microsystems). The following substitution sequence was used: −90 °C for 48 h, +10 °C increment/hour for 6 h, −30 °C for 20 h, 5 °C increment/hour for 9 h, and 15 °C for 30 min. Substituted samples were then progressively exchanged in increasing concentrations of low viscosity Epon resin. Samples permeated in 100% resin were embedded in a flat layer of resin and polymerized at 60 °C for 12 h. Flat embedded samples were observed and selected under a light microscope before serial semi-thick sectioning (300 nm) using a UC6 Ultramicrotome (Leica Mycrosystem, Austria). Serial sections were collected on Formvar-coated EM slot grids and post-stained in 2% Uranyl Acetate in 70% Methanol for 15 min followed by Reynold’s lead citrate for 5 min. 10 nm colloidal gold particles were deposited on both surfaces of the sections. Dual axis electron tomography was performed on a Tecnai F30 microscope (FEI, The Netherlands) operated at 300 kV with a US1000 CCD camera (Gatan, USA). Images were collected over a ±60° range at 1° increment using the SerialEM software^30^. The samples were acquired at a magnification of 4700× with pixel size 2.32 nm. Five tilted serial sections per spindle were reconstructed using the IMOD software package (bio3d.colorado.edu/imod/) with the gold particles as fiducial markers. The Amira software (FEI, The Netherlands) was used for the segmentation and automated tracing of microtubules. Stitching of the 5 serial tomogram slices and their corresponding microtubule traces was performed in ZIBAmira^29,31^. Automatic microtubule tracing was then visually inspected and manually edited for potential tracing errors or omissions. Chromosome masses were segmented manually in Amira. Microtubule lengths and orientations were directly extracted from Amira. We determined the average tilts of the reconstructed spindles relative to the *Y* and *Z* axes as the mean angles of central spindle microtubules relative to these axes. Microtubule overlaps were calculated using a custom program written in C and assuming that microtubules distant from <45 nm were overlapping microtubules. The position and length of each overlap was extracted from microtubules after segmentation in both reconstructions. To exclude false overlaps caused by microtubules crossing at a steep angle, we calculated the minimal expected overlap length for microtubules distant from <45 nm included in a 30° cone (more than 80% of central spindle microtubules will be contained within this cone, see Fig. 2b, d). Simple trigonometry gives Min(Overlap) = 2x(45/Tan(30°)) = 156 nm. We conservatively fixed the minimal microtubule overlap length at 200 nm.

### Graphs and statistical analysis

Experiments were repeated at least twice and a minimum number of 6 embryos were quantified for each experimental condition. All graphs and statistical analysis were done with GraphPad Prism 6 (GraphPad Software). Statistical significance was evaluated using unpaired *t*-tests with Welch’s correction, or one-way ANOVA with Dunnett’s, Tukey’s, or Holm-Sidak’s multiple comparison tests.

### Data availability

The data that support the findings of this study are available from the corresponding author upon request.

## Electronic supplementary material

Supplementary Information

Description of Additional Supplementary Files

Supplementary Movie 1

Supplementary Movie 2

Supplementary Movie 3

Supplementary Movie 4

Supplementary Movie 5

Supplementary Movie 6

Supplementary Movie 7

Supplementary Movie 8
