# Supplementary material for: Chromosome segregation occurs by microtubule pushing in oocytes
Source: Nat Commun. 2017 Nov 14;8:1499. doi: 10.1038/s41467-017-01539-8 (PMC5684144; doi:10.1038/s41467-017-01539-8)
Supplement: Supplementary file 1 — Supplementary Information [file 41467_2017_1539_MOESM1_ESM.pdf]

a

Distance and timings of events in 30 control fertilized oocytes +/- SD		
	Distance between chromosomes	Time after anaphase I onset
Anaphase A-B transition:	2.37 μm +/- 0.27	193 s +/- 60
Polar body extrusion start:	3.03 μm +/- 0.39	246 s +/- 54

b

Number of microtubules contained whithin the indicated populations

		Poles	Central array	Chromosomal array - Both	Chromosomal array - PB	Chromosomal array - PN	Total	Total combined MT length
Mid anaphase I	All MTs	1225	963	12	250	430	2880	1499 μm
	% uncut MTs	58.8	44.6	33.3	55.6	51.2	52.5	
Late anaphase I	All MTs	81	537	6	314	372	1310	1102 μm
	% uncut MTs	61.7	45.4	83.3	51.6	70.1	55.1	

c

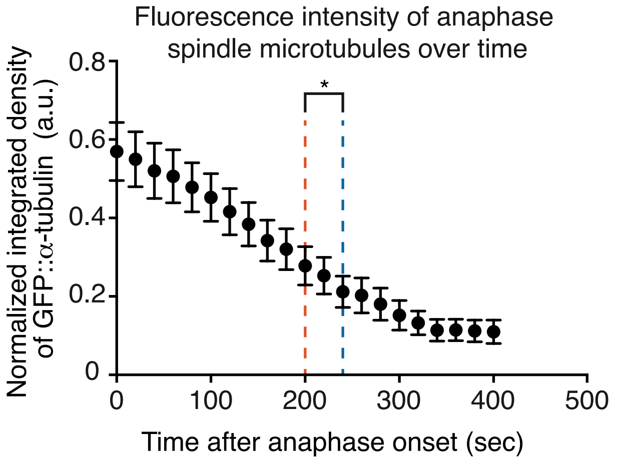

d

Analysis of spindle fluorescence intensity		
Time (sec)	Normalized integrated density +/- SD	n =
200	0.28 +/- 0.13	31
240	0.21 +/- 0.11	31

e

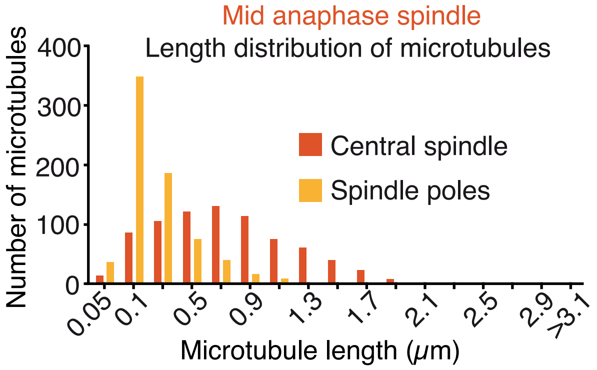

f

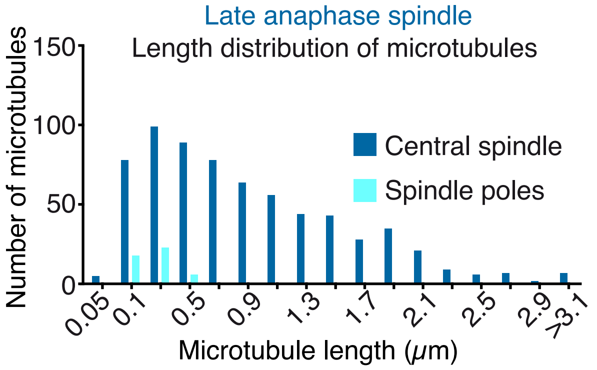

g

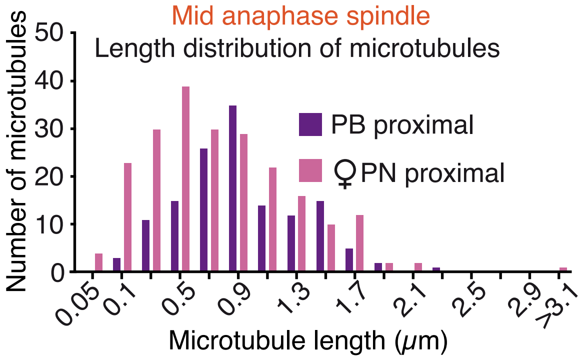

h

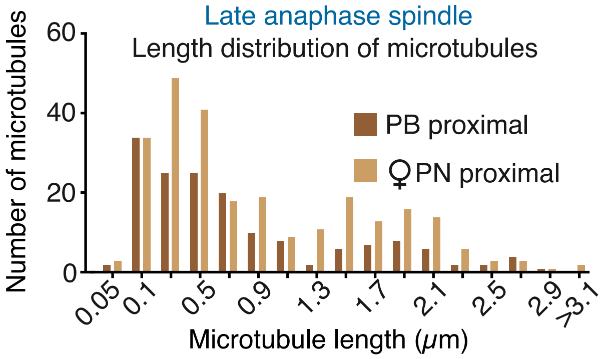

Supplementary Figure 1: Analysis of the various microtubule populations in the reconstructed spindles. (a) Distance between the segregating chromosomes and time after anaphase onset for the indicated meiotic events. **(b)** Distribution of the different microtubule populations in each reconstructed spindle (top rows) and the proportion of uncut fully contained microtubules within the partial reconstructions for each population (bottom rows). The relative distribution between the different microtubule populations was not significantly altered by the partial reconstruction. **(c)** Quantification of the normalized integrated spindle fluorescence over time. Error bars indicate 95% CI. Difference between intensities at 200 and 240s, $p=0.0364$ using unpaired t-test with Welch's correction. Estimated timing for early and late EM reconstructions is indicated in red and blue respectively. **(d)** Quantification of the normalized integrated spindle fluorescence density (±SD) for the indicated time points. **(e)** Microtubule length distribution in the mid-anaphase I spindle. **(f)** Microtubule length distribution in the late anaphase I spindle. **(g)** Microtubule length distribution in the mid-anaphase I chromosomal arrays. **(h)** Microtubule length distribution in the late anaphase I chromosomal arrays.

a

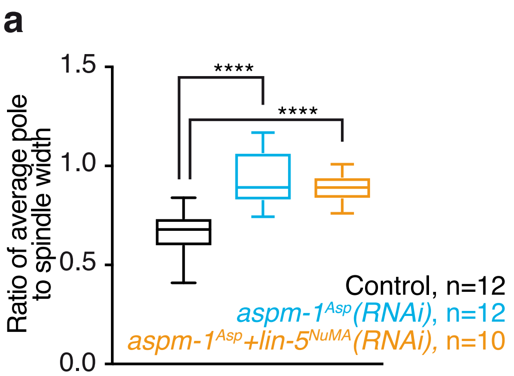

Analysis of spindle bipolarity

	Pole width average (μm)	Spindle width average (μm)	Spindle length average (μm)
Control:	3.94 +/- 0.54	5.96 +/- 0.54	6.25 +/- 0.98
aspm-1[Asp]*(RNAi):*	4.86 +/- 0.68	5.32 +/- 1.07	9.15 +/- 1.43
aspm-1[Asp]*+lin-5*[NuMA]*(RNAi):*	4.55 +/- 0.47	5.14 +/- 0.73	8.12 +/- 2.2

b

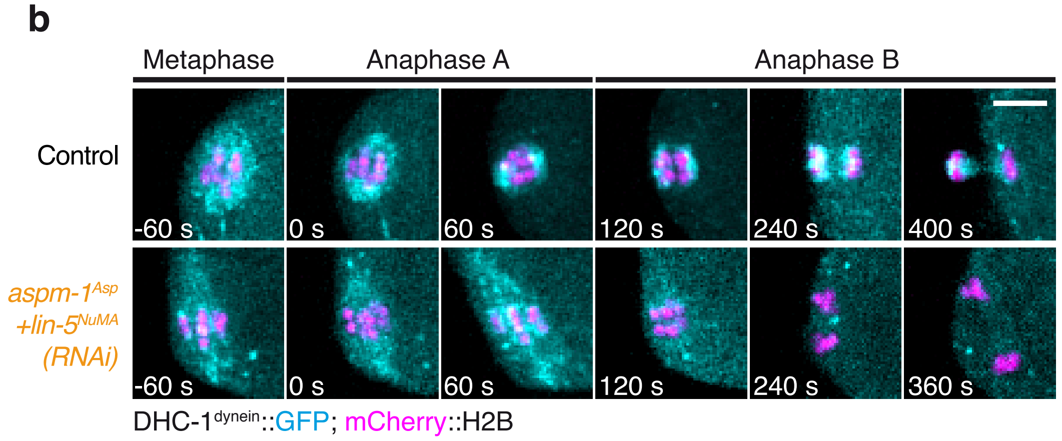

c

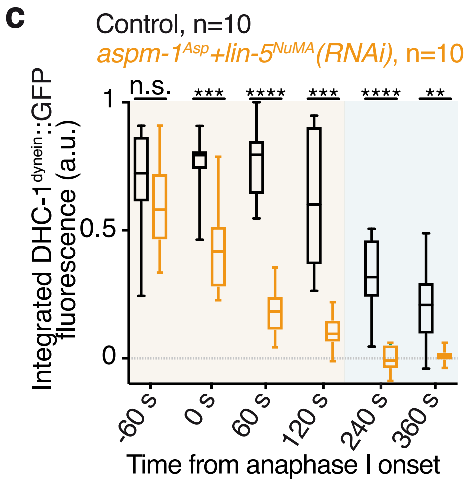

Supplementary Figure 2: Meiotic chromosome segregation occurs in absence of dynein on or around chromosomes when ASPM-1^{Asp} and LIN-5^{NuMA} are depleted. **(a)** Quantification of the average pole-to-spindle width ratios (±SD) for the indicated conditions. The table on the right also includes the quantification of spindle lengths in each condition. (One-way ANOVA: *aspm-1* ^{Asp}*(RNAi)*, p<0.0001; *aspm-1*^{Asp}*+lin-5* ^{NuMA}*(RNAi)*, p<0.0001) **(b)** Still images from live imaging of GFP-tagged DHC-1^{dynein} and mCherry-tagged histone H2B expressing fertilized oocytes in the indicated conditions. Timings are indicated at the bottom left corner of each frame. Scale bar, 5 μm. **(c)** Quantification of the normalized GFP-tagged DHC-1^{dynein} integrated fluorescence intensity around chromosomes over time in the indicated conditions. Background colors represent average duration of anaphase A (light orange) and B (light blue) respectively. Error bars represent 1-99 percentile of data. (Unpaired t-test with Welch's correction: *aspm-1*^{Asp}*+lin-5* ^{NuMA}*(RNAi)* at -60s, p=0.2418; 0s, p=0.0001; 60s, p<0.0001; 120s, p=0.0002; 240s, p<0.0001; spindle breaks, p=0.0027)

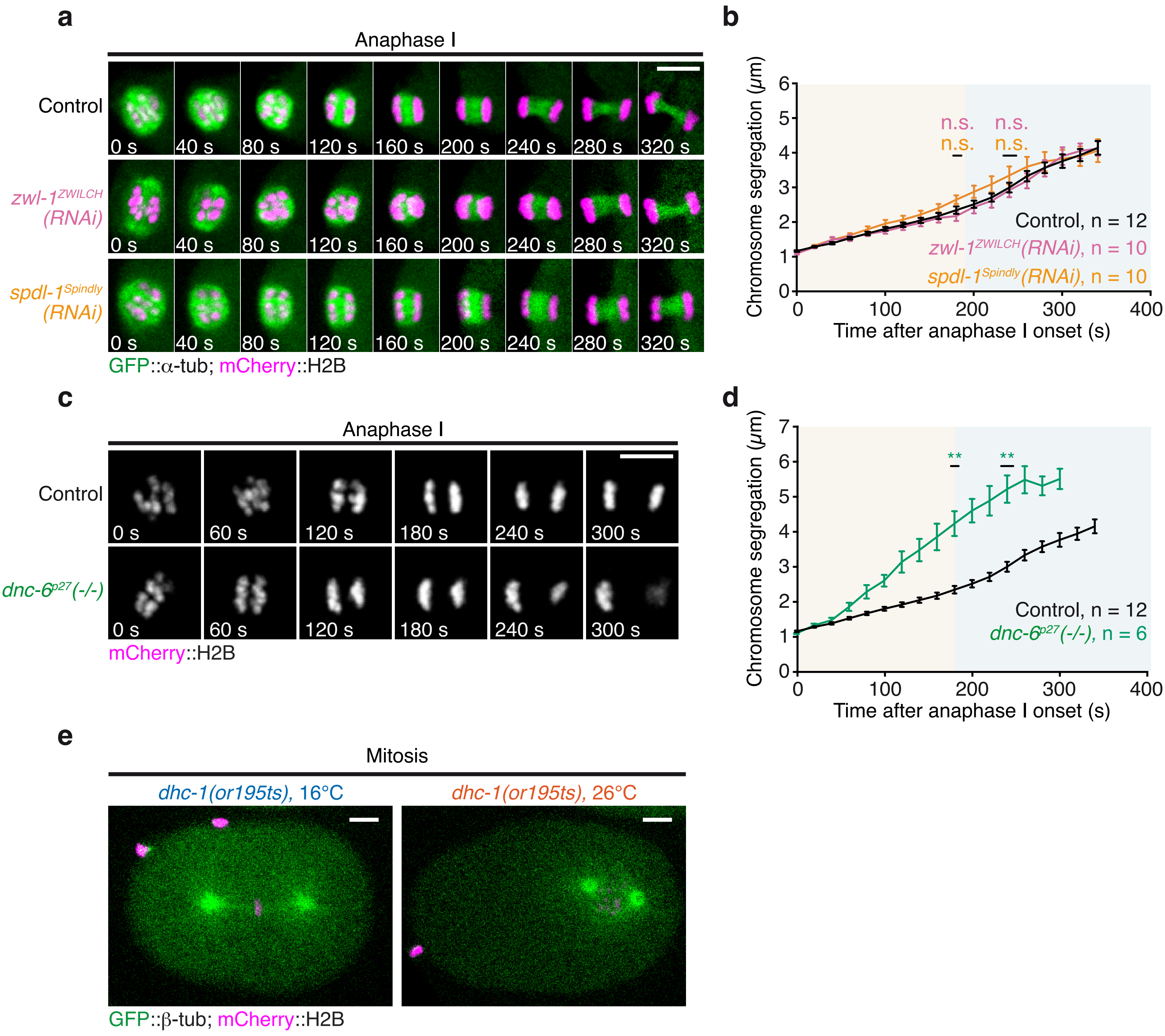
a
Anaphase I
Control
zwl-1^ZWILCH (RNAi)
spdl-1^Spindly (RNAi)
0 s
40 s
80 s
120 s
160 s
200 s
240 s
280 s
320 s
GFP::α-tub; mCherry::H2B
b
Chromosome segregation (µm)
Time after anaphase I onset (s)
n.s.
n.s.
n.s.
n.s.
Control, n = 12
zwl-1^ZWILCH(RNAi), n = 10
spdl-1^Spindly(RNAi), n = 10
c
Anaphase I
Control
dnc-6^p27(-/-)
0 s
60 s
120 s
180 s
240 s
300 s
mCherry::H2B
d
Chromosome segregation (µm)
Time after anaphase I onset (s)
**
**
Control, n = 12
dnc-6^p27(-/-), n = 6
e
Mitosis
dhc-1(or195ts), 16°C
dhc-1(or195ts), 26°C
GFP::β-tub; mCherry::H2B

Supplementary Figure 3: Dynein and its kinetochore-targeting proteins are not required for meiotic chromosome segregation. **(a)** Still images from live imaging of fertilized oocytes expressing GFP-tagged α-tubulin and mCherry-tagged histone H2B in the indicated conditions. Timings from anaphase onset are indicated at the bottom of each frame. Scale bar, 5 μm. **(b)** Distance between the centroid of each set of segregating chromosomes over time after anaphase onset in the indicated conditions. Tracking was performed until the anaphase spindle broke apart at the end of polar body extrusion. Error bars represent the SEM. Background colors represent average duration of anaphase A (light orange) and B (light blue) respectively. (One-way ANOVA: *zwl-1*^{ZWILCH}*(RNAi)* at 180 s, p=0.9996; *spdl-1* ^{Spindly}*(RNAi)* at 180 s, p=0.9884; *zwl-1* ^{ZWILCH}*(RNAi)* at 240 s, p=0.9998; *spdl-1* ^{Spindly}*(RNAi)* at 240 s, p=0.9662) **(c)** Still images from live imaging of fertilized oocytes expressing mCherry-tagged histone H2B in the indicated conditions. Timings from anaphase onset are indicated at the bottom of each frame. Scale bar, 5 μm. **(d)** Distance between the centroid of each set of segregating chromosomes over time after anaphase onset in the indicated conditions. Tracking was performed until the anaphase spindle broke apart at the end of polar body extrusion. Error bars represent the SEM. Background colors represent average duration of anaphase A (light orange) and B (light blue) respectively. (Unpaired t-test with Welch's correction: *dnc-6*^{p27}*(-/-)* at 180 s, p=0.0087; *dnc-6*^{p27}*(-/-)* at 240 s, p=0.0093) **(e)** Still images from live imaging of early embryos expressing GFP-tagged β-tubulin and mCherry-tagged histone H2B in the indicated conditions. Scale bar, 5 μm.

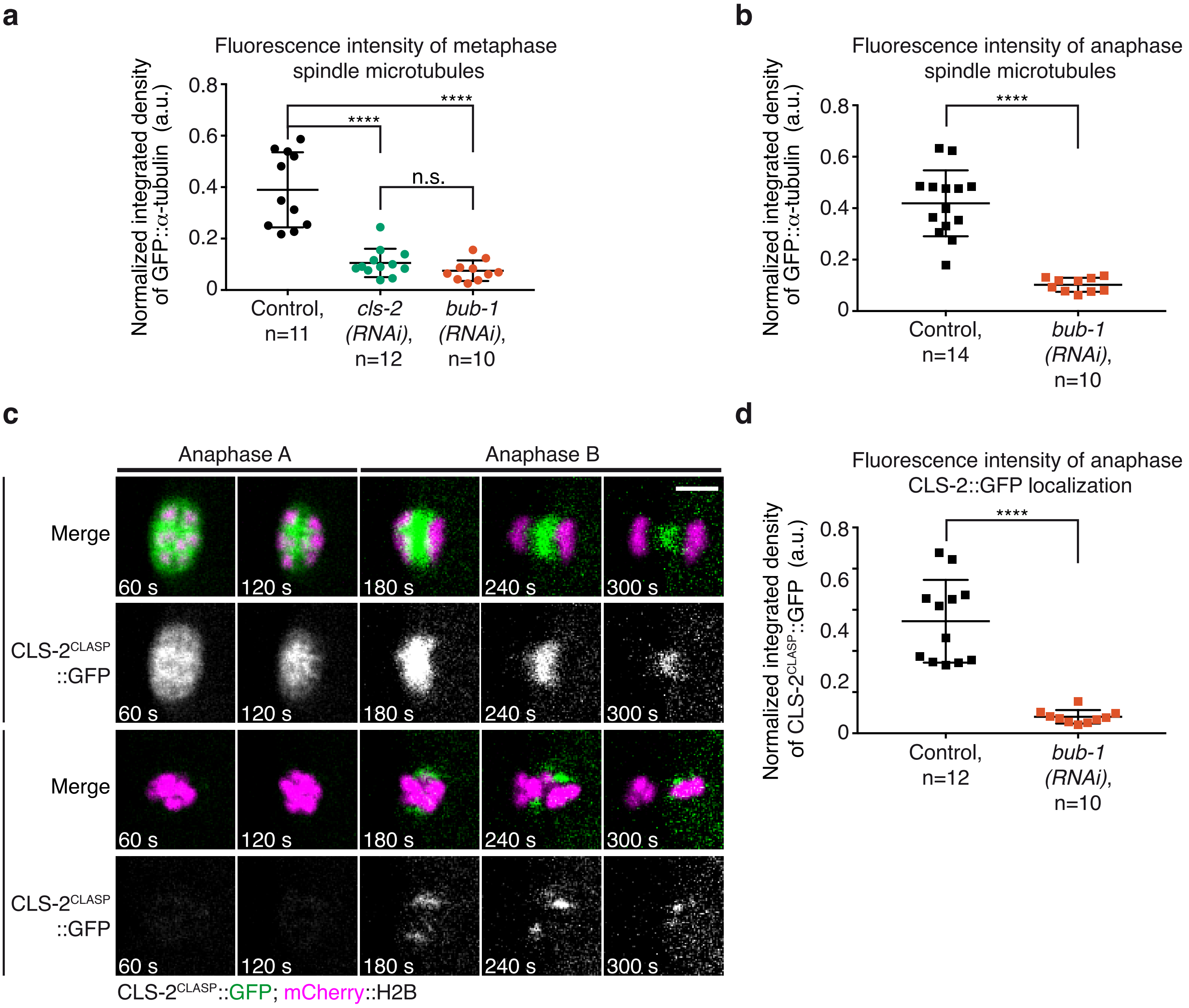
a
Fluorescence intensity of metaphase spindle microtubules
Normalized integrated density of GFP::α-tubulin (a.u.)
0.8
0.6
0.4
0.2
0
****
****
n.s.
Control, n=11
cls-2 (RNAi), n=12
bub-1 (RNAi), n=10
b
Fluorescence intensity of anaphase spindle microtubules
Normalized integrated density of GFP::α-tubulin (a.u.)
0.8
0.6
0.4
0.2
0
****
Control, n=14
bub-1 (RNAi), n=10
c
Anaphase A
Anaphase B
Control
Merge
CLS-2CLASP ::GFP
bub-1(RNAi)
Merge
CLS-2CLASP ::GFP
60 s
120 s
180 s
240 s
300 s
CLS-2CLASP::GFP; mCherry::H2B
d
Fluorescence intensity of anaphase CLS-2::GFP localization
Normalized integrated density of CLS-2CLASP::GFP (a.u.)
0.8
0.6
0.4
0.2
0
****
Control, n=12
bub-1 (RNAi), n=10

Supplementary Figure 4: BUB-1 and CLS-2^{CLASP} depletions lead to similar pre-anaphase spindle defects and CLS-2^{CLASP} localization is evident in conditions where segregation occurs. **(a)** Quantification of the normalized integrated spindle fluorescence before anaphase for the indicated conditions. Error bars indicate SD. (One-way ANOVA: Control vs *cls-2*^{CLASP}*(RNAi)*, p<0.0001; Control vs *bub-1(RNAi)*, p<0.0001; *cls-2* ^{CLASP} *(RNAi)* vs *bub-1(RNAi)*, p=0.7308) **(b)** Quantification of the normalized integrated spindle fluorescence at the onset of polar body extrusion for the indicated conditions. Error bars indicate SD. (Welch's t-test: Control vs *bub-1(RNAi)*, p<0.0001) **(c)** Still images from live imaging of fertilized oocyte expressing GFP-tagged CLS-2^{CLASP} and mCherry-tagged histone H2B in the indicated conditions. Each time point of the control oocyte was scaled independently. Time points of the BUB-1-depleted oocyte were scaled according to the corresponding control time point. Timings from anaphase onset are indicated at the bottom left corner of each frame. Scale bar, 5 μm. **(d)** Quantification of the normalized integrated CLS-2^{CLASP}::GFP fluorescence at the onset of polar body extrusion for the indicated conditions. Error bars indicate SD. (Welch's t-test: Control vs *bub-1(RNAi)*, p<0.0001)

a

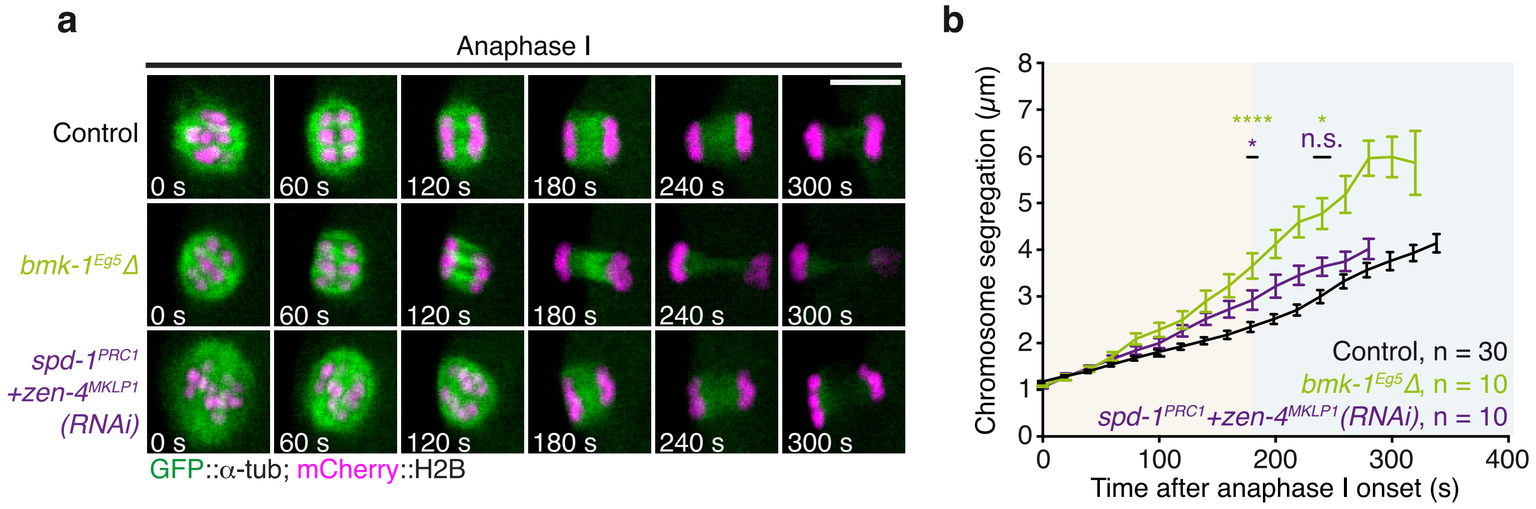

c

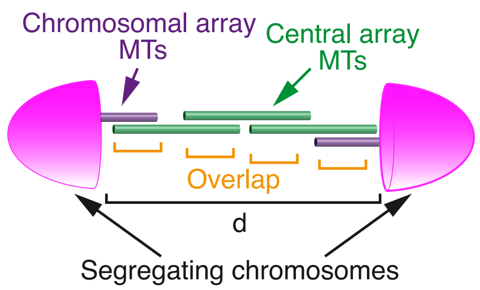

$$d=(2\times L_{chr})+(nMTs\times L_{cent})-(nMTs+1)\times L_{over}$$

$$nMTs=(d-2\times L_{chr}+L_{over})/(L_{cent}-L_{over})$$

	Chromosomal array MTs (L_{chr})	Central array MTs (L_{cent})	MT overlap (L_{over})	Distance between the segregating chromosomes (d)	Number of central MTs linking the chromosomal array (nMTs)
Mid anaphase I	508 nm	665 nm	446 nm	1750 nm	5.4
Late anaphase I	848 nm	956 nm	552 nm	2710 nm	3.9

Supplementary Figure 5: Neither the kinesin-5 BMK-1^{Eg5} nor the kinesin-6 ZEN-4^{MKLP1} and its partner microtubule-associated protein SPD-1^{PRC1} are required for meiotic chromosome segregation. Model of meiotic chromosome segregation by pushing. (a) Still images from live imaging of fertilized oocyte expressing GFP-tagged α-tubulin and mCherry-tagged histone H2B in the indicated conditions. Timings from anaphase onset are indicated at the bottom left corner of each frame. Scale bar, 5 μm. **(b)** Distance between the centroid of each set of segregating chromosomes over time after anaphase onset. Tracking was performed until the anaphase spindle broke apart at the end of polar body extrusion. Error bars represent the SEM. Background colors represent average duration of anaphase A (light orange) and B (light blue) respectively. (One-way ANOVA: *bmk-1^{Eg5}(RNAi)* at 180 s, $p<0.0001$; *spd-1^{PRC1}+zen-4^{MKLP1}(RNAi)* at 180 s, $p=0.0237$; *bmk-1^{Eg5}(RNAi)* at 240 s, $p=0.0134$; *spd-1^{PRC1}+zen-4^{MKLP1}(RNAi)* at 240 s, $p=0.7037$) **(c)** Calculation and numerical values used for estimating the number of overlapping microtubules required to travel the distance that separates the segregating chromosomes in mid- and late anaphase I.

Gene	Primer 1*	Primer 2*	Template	Final [C] (µg/µl)
R107.6 *(cls-2)*	5’-taatacgactcactataggTTCAAGGAAAAGTTGGACC-3’	5’-aattaaccctcactaaaggGGTGCATTTCTGATTCCACC-3’	*cls-2* cDNA (pJD122)	1.7
T09A5.10 *(lin-5)*	5’-taatacgactcactataggGCTTCATTACCACACTTGCG-3’	5’-aattaaccctcactaaaggATCGCCGAGGAGGCACAATT-3’	N2 cDNA	1.5
C45G3.1 *(aspm-1)*	5’-taatacgactcactataggTGGGTGTTCCAACGGATAAT-3’	5’-aattaaccctcactaaaggGCTTCAAGAACTACGACGCC-3’	N2 cDNA	1
M03D4.1 *(zen-4)*	5’-taatacgactcactataggATTGGAGCTGTTGGATGAGC-3’	5’-aattaaccctcactaaaggAATTGGTTATGGCTCCGAGA-3’	*zen-4* cDNA (pJD215)	1.5
Y34D9A.4 *(spd-1)*	5’-taatacgactcactataggCTCTTCCCAGTAAAGGCGTTCG-3’	5’-aattaaccctcactaaaggTTTAGCCACGGGCTCCATCTTCG-3’	N2 genomic DNA	1.5
Y39G10AR.2 *(zwl-1)*	5’-taatacgactcactataggGGATCAGTGAAGCGAGATGACTC-3’	5’-aattaaccctcactaaaggATGCCACTCACCATCGAGCAG-3’	N2 cDNA	1.5
C06A8.5 *(spdl-1)*	5’-taatacgactcactataggCCTAATTGAGGCATGGGTTC-3’	5’-aattaaccctcactaaaggAACGTTACCCGAATGACCAC-3’	N2 genomic DNA	1.2
T21E12.4 *(dhc-1)*	5’-taatacgactcactataggCCTTTCCTTCCTGGGTCTTC-3’	5’-aattaaccctcactaaaggAAGGAAGGAGCTCAACGACA-3’	N2 genomic DNA	1.2
R06C7.8 *(bub-1)*	5’-taatacgactcactataggTCTGAGATTCTTCCGGTTCG-3’	5’-aattaaccctcactaaaggTGCCAAATGGAAGGACACTT-3’	N2 genomic DNA	1.5
R06C7.8 *(bub-1)*	5’-taatacgactcactataggCTACTTTTGGTTGGCGGCAAG-3’	5’-aattaaccctcactaaaggGGATAATTTTATGATCACCAG-3’	*bub-1* cDNA (pJD42)	1

Supplementary Table 1: *C. elegans* strains used in this study

*Lowercase letters denote T3 and T7 sequences included for RNA synthesis

Strain	Genotype
N2	*Wild Type (Ancestral N2 Bristol)*
JCC483	*ojIs1[pie-1::GFP::tbb-2::tbb-2]; unc-119(ed3) III?; ltIs37[pAA64; pie-1/mCHERRY::his-58; unc-119 (+)]IV*
OD203	*orls17[dhc-1::dhc-1::GFP;cb-unc- 119(+)]; ruIs32[pAZ132; pie-1::mCherry::his-58], unc-119(ed3)?III*
JDU107	*ijmSi3[pJD342; mex-5::cls-2reenc::GFP::tbb-2; cb-unc-119(+)]I; ltSi264[pTK011; Pbub-1::bub-1reenc::mCherry::bub-1; cb-unc-119(+)]II; unc-119(ed3)III?*
JDU146	*ijmSi3[pJD342; mex-5::cls-2reenc::GFP::tbb-2; cb-unc-119(+)]I;; ijmSi31[pJD446; mex-5::mCherry::his-11::tbb-2]II; unc-119(ed3)III?*
JDU205	*ijmSi53[pJD471;mex-5::cls-2reenc-W57A_K177A_R224A::GFP::tbb-2; cb-unc-119(+)]I; ijmSi31[pJD446_Pmex-5::mCherry::his-11::tbb-2]II; unc-119(ed3)III?*
JDU233	*ijmSi63[pJD520; mex-5::GFP::tba-2; mCherry::his-11; cb-unc-119(+)]II; unc-119(ed3)III?*
JDU282	*ijmSi53[pJD471;mex-5::cls-2reenc-W57A_K177A_R224A::GFP::tbb-2; cb-unc-119(+)]I;ijmSi63[pJD520; mex-5::GFP::tba-2; mCherry::his-11; cb-unc-119(+)]II; unc-119(ed3)III?*
JDU405	*ijmSi63 [pJD520; mosII_5'mex-5_GFP::tba-2; mCherry::his-11; cb-unc-119(+)] II; unc-119(ed3) III?; bmk-1(ok391)V*
JDU243	*ijmSi3 [pJD342/pJD330; ChrI_5'mex-5_cls-2reenc::GFP_tbb-2; cb-unc-119(+)] I; ijmSi63 [pJD520; mosII_5'mex-5_GFP::tba-2; mCherry::his-11; cb-unc-119(+)] II; unc-119(ed3)III?*
GCP485	*dnc-6[prt48(knockout/C-terminal 3xflag)] I; ltIs37[pAA64; pie-1/mCherry::his-58; unc-119 (+)] IV; mcm-4(e1466) dpy-5(e61) I/hT2 [bli-4(e937) let-?(q782) qIs48] (I;III)*
JCC912	*dhc-1(or195ts)I; ojIs1[pie-1::GFP::tbb-2::tbb-2]; unc-119(ed3) III?; ltIs37[pAA64; pie-1/mCHERRY::his-58; unc-119 (+)]IV*

Supplementary Table 2: Primers and templates used for dsRNA production

**[unc-119(ed3)?]* was present in the parental strains, but these strains have not been directly sequenced to determine if the *unc-119* gene contains the *ed3* mutation.
