# Supplementary material for: Chromosome segregation occurs by microtubule pushing in oocytes
Source: Nat Commun. 2017 Nov 14;8:1499. doi: 10.1038/s41467-017-01539-8 (PMC5684144; doi:10.1038/s41467-017-01539-8)
Supplement: Supplementary file 2 — Description of Additional Supplementary Files [file 41467_2017_1539_MOESM2_ESM.pdf]

Description of Additional Supplementary Files

File Name: Supplementary Movie 1

Description: Partial reconstruction of a mid-anaphase I spindle from a C. elegans oocyte. 3D projection of the segmented chromosomes (magenta) and traced microtubules (green), followed by dual axis rotations, and a recapitulation of the microtubules contained in the poles (dark grey) as well as chromosome proximal (yellow and orange) and central spindle (teal) arrays.

File Name: Supplementary Movie 2

Description: Partial reconstruction of a late anaphase I spindle from a C. elegans oocyte. 3D projection of the segmented chromosomes (magenta) and traced microtubules (green), followed by dual axis rotations, and a recapitulation of the microtubules contained in the poles (dark grey) as well as chromosome proximal (yellow and orange) and central spindle (teal) arrays.

File Name: Supplementary Movie 3

Description: Movie montage of anaphase I in GFP::α-tubulin (green) and mCherry::H2B (magenta) expressing fertilized oocytes in control (top), aspm-1Asp(RNAi) (second from top), aspm-1Asp+lin-5NuMA(RNAi) (second from bottom), or aspm-1Asp+dhc-1dynein(RNAi)(bottom). Images, which are the maximum projection of 4 z-sections, were collected every 20 seconds and played back at 120x real time (12 images per second) with time 0 corresponding to anaphase onset. Scale bar, 5 μm.

File Name: Supplementary Movie 4

Description: Movie montage of anaphase I in DHC-1dynein::GFP (cyan) and mCherry::H2B (magenta) expressing fertilized oocytes in control (top) or aspm-1Asp+lin-5NuMA(RNAi) (bottom). Images, which are the maximum projection of 4 z-sections, were collected every 20 seconds and played back at 120x real time (12 images per second) with time 0 corresponding to anaphase onset. Scale bar, 5 μm.

File Name: Supplementary Movie 5

Description: Movie montage of anaphase I in GFP::α-tubulin (green) and mCherry::H2B (magenta) expressing fertilized oocytes in control (top), zwl-1ZWILCH(RNAi) (middle), or spdl-1Spindly(RNAi)(bottom). Images, which are the maximum projection of 4 z-sections, were collected every 20 seconds and played back at 120x real time (12 images per second) with time 0 corresponding to anaphase onset. Scale bar, 5 μm.

File Name: Supplementary Movie 6

Description: Movie montage of anaphase I in GFP:: β-tubulin (green) and mCherry::H2B (magenta) expressing fertilized oocytes in control embryos at 26°C (top), dhc-1(or195ts) at 16°C (second from top), dhc-1(or195ts) at 26°C (second from bottom), or aspm-1Asp+lin-5NuMA(RNAi) in dhc-1(or195ts) at 26°C (bottom). Images, which are the maximum projection of 4 z-sections, were collected every 20 seconds and played back at 120x real time (12 images per second) with time 0 corresponding to anaphase onset. Scale bar, 5 μm.

File Name: Supplementary Movie 7

Description: Movie montage of anaphase I in GFP:: α-tubulin (green) and mCherry::H2B (magenta) expressing fertilized oocytes in control (top), cls-2CLASP (RNAi) (middle), and GFP:: α-Tubulin (green), CLS-2CLASP 3A::GFP (green) and mCherry::H2B (magenta) expressing fertilized oocyte with cls-2CLASP(RNAi) (bottom). Images, which are the maximum projection of 4 z-sections, were collected every 15 seconds and played back at 90x real time (12 images per second) with time 0 corresponding to anaphase onset. Scale bar, 5 μm.

File Name: Supplementary Movie 8

Description: Movie montage of anaphase I in GFP:: α-Tubulin (green) and mCherry::H2B (magenta) expressing fertilized oocytes in control (top), bmk-1Eg5 Δ mutant (middle) or spd-1PRC1+zen-4MKLP1(RNAi) (bottom). Images, which are the maximum projection of 4
z-sections, were collected every 20 seconds and played back at 120x real time (12 images per second) with time 0 corresponding to anaphase onset. Scale bar, 5 μm.
